# *Streptococcus suis* Outbreak with Symptoms of Toxic Shock Syndrome in China

**DOI:** 10.1371/journal.pmed.0030218

**Published:** 2006-04-11

*Streptococcus suis* is a pathogen with serious economic effects on the pig industry worldwide. The disease is endemic in adult pigs in most countries where pig farming is common. Infections in adult pigs are usually asymptomatic, but infant piglets that get infected through contact with colonized adult females can develop fatal sepsis. Transmission to humans is rare and generally restricted to individuals with occupational exposure to live or dead pigs. The first human case of *S. suis* infection was reported in Denmark in 1968. Most of the approximately 200 previously reported human cases were characterized by meningitis and septicemia, and associated with a mortality of less than 10%.

Now George Gao and colleagues from the Chinese Academy of Sciences, the Chinese Center for Disease Control and Prevention, and other Chinese institutions report details of a recent unusual outbreak in 2005 of *S. suis* that affected over 200 individuals in Sichuan province and killed 38 of them. Besides the large number of individuals infected and the high mortality rate, it was the clinical symptoms associated with this outbreak that attracted interest and worry from scientists and health officials worldwide when the outbreak was first reported in the news last year.

As Gao and colleagues now detail in their article, a large proportion of the individuals infected (including all but one of the patients who died) showed symptoms of streptococcal toxic shock syndrome (STSS). Up to now, most reported cases of STSS had been attributed to group A streptococci. However, as Gao and colleagues show, the etiologic agents in the recent outbreak, as well as in an earlier outbreak in Sichuan province in 1998 that killed 14 of 25 reported patients, were clearly of the serotype 2 strains of *S. suis*. Both human outbreaks were closely linked to outbreaks in the local pig populations, and the researchers report that there are no reasons to believe that any of the cases had been caused by human-to-human transmission. They also showed that *S. suis* isolated from the human patients caused typical *S. suis* disease in newborn piglets.

Were there other unusual characteristics among the Chinese isolates that could explain their ability to cause STSS? STSS is thought to be caused by bacterial superantigens that overstimulate the human immune system. Gao and colleagues tested the *S. suis* isolates associated with STSS for superantigen production, but were unable to detect any.

One of the key questions that arose when the recent outbreak was first reported was whether a new and more virulent strain of *S. suis* has emerged in China. Gao and colleagues performed an initial genomic survey of the isolates from the Chinese outbreaks to look for unusual characteristics that could explain the virulence of the pathogens. They did find some differences between the isolates from the 1998 and 2005 Chinese outbreaks (which appear very similar to each other) and other virulent strains, but a more detailed sequence analysis and functional studies will be needed before it is clear whether any of these differences have functional consequences for pathogenesis in pigs or humans.

In an accompanying Perspective article ( 10.1371/journal.pmed.0030187), Shiranee Sriskandan and Joshua Slater suggest that *S. suis* infection “should now be in the list of differential diagnoses when clinicians encounter patients with unexplained sepsis who have a history of exposure to pigs.” They conclude that “the emergence of any new zoonotic disease associated with high mortality is of global concern” and call for “international collaboration … to clarify differences between isolates circulating in different regions of the world.”

